# Histone deacetylase enzymes and selective histone deacetylase inhibitors for antitumor effects and enhancement of antitumor immunity in glioblastoma

**DOI:** 10.20517/2347-8659.2018.58

**Published:** 2018-11-12

**Authors:** Caleb J. Yelton, Swapan K. Ray

**Affiliations:** Department of Pathology, Microbiology, and Immunology, University of South Carolina School of Medicine, Columbia, SC 29209, USA.

**Keywords:** Glioblastoma, histone deacetylase inhibitors, antitumor effects, antitumor immunity

## Abstract

Glioblastoma multiforme (GBM), which is the most common primary central nervous system malignancy in adults, has long presented a formidable challenge to researchers and clinicians alike. Dismal 5-year survival rates of the patients with these tumors and the ability of the recurrent tumors to evade primary treatment strategies have prompted a need for alternative therapies in the treatment of GBM. Histone deacetylase (HDAC) inhibitors are currently a potential epigenetic therapy modality under investigation for use in GBM with mixed results. While these agents show promise through a variety of proposed mechanisms in the pre-clinical realm, only several of these agents have shown this same promise when translated into the clinical arena, either as monotherapy or for use in combination regimens. This review will examine the current state of use of HDAC inhibitors in GBM, the mechanistic rationale for use of HDAC inhibitors in GBM, and then examine an exciting new mechanistic revelation of certain HDAC inhibitors that promote antitumor immunity in GBM. The details of this antitumor immunity will be discussed with an emphasis on application of this antitumor immunity towards developing alternative therapies for treatment of GBM. The final section of this article will provide an overview of the current state of immunotherapy targeted specifically to GBM.

## INTRODUCTION

Glioblastoma multiforme (GBM) is the most prevalent primary malignancy in the central nervous system (CNS) in adults. GBM still remains incurable and thus continues to present a formidable challenge to both clinicians and researchers alike. Classified as a grade IV glioma by the World Health Organization (WHO)^[1]^ this tumor’s dismal survival rates are owed to its ability to recur following first-line treatment strategies such as surgical resection, radiation therapy, and chemotherapeutic agents - the current standard of care. This tumor also possesses the ability to evade current first-line treatment strategies through the development of multiple resistance mechanisms, which it employs to recur despite initial response to these strategies^[2]^. GBM is also simply called glioblastoma. The most resistant glioblastoma cells, also known as glioblastoma stem cells (GSCs), which remain alive following first-line therapy have employed resistance mechanisms and will go on to form recurrent glioblastomas. These tumors are more difficult to treat as they confer resistance to first-line treatment strategies, requiring alternative therapies. The agents currently needed to combat these recurrent glioblastomas are lacking. As such, developing novel therapeutic agents based on inquiries into the biochemical specificities and pathogeneses of these tumors has been a hotbed of research in recent years. Novel therapeutic agents have shown considerable promise in their developmental phases but have yet to replace the current standard of care. The current standard of care includes surgical resection, radiotherapy, and the chemotherapy using temozolomide (TMZ)^[3]^. Multiple avenues have been explored for potential therapeutic strategies. One particularly exciting avenue of research is immunotherapy, which harnesses the immune system to aid in abolishing the growth of glioblastoma^[4,5]^.

Immunotherapy has seen success in the clinical realm in recent years, a success that can be attributed to a more robust understanding of basic tumor immunology in order to aid the immune system in fighting a neoplastic process^[6]^. Previously, the lack of clinical efficacy in immunotherapy was due to the ability of many tumors to avoid recognition and therefore elimination by the immune system^[7]^. However, active research in this area into how the tumor evades the immune system has led to novel therapies in the fight against these pathologies, with cancer immunotherapy even being heralded as the “breakthrough of the year” roughly five years ago^[8]^. Most recently, immunotherapy specific to malignancies has been such an exciting breakthrough that Drs. James P. Allison and Tasuku Honjo have received the 2018 Nobel Prize in Physiology or Medicine for their contributions to the field of cancer immunotherapy and identification of the immune checkpoint proteins [e.g., programmed death-1 (PD-1)/programmed death-ligand 1 (PD-L1), cytotoxic T lymphocyte associated antigen-4 (CTLA-4)] that usually act as a brake on the immune system. For modulating the immune system, these therapies have employed multiple strategies including inhibiting immune checkpoints, expanding an existing immune system response, enhancing the immunological profile of solid tumors, natural killer (NK) cell/chimeric antigen receptor (CAR)-T cell modulation, and T regulatory (Treg)/myeloid suppressor cell modulation^[9]^. Immunotherapy, like any other new treatment modality heralded as panacea, ultimately has its limitations and downsides when being used to treat cancer. These limitations are especially evident with glioblastoma, as certain modalities of cancer immunotherapy (immune checkpoint inhibitors, CAR-T cell therapy, *etc*.) require continued research and further clinical trials if they are to be considered in the next step in the targeted glioblastoma therapy^[10,11]^.

A challenge specific to glioblastoma and a potential barrier to the application of immunotherapy to these tumors is the presence of the blood-brain barrier (BBB), which forms a protective coating around the brain made up of tight junctions between astrocytes. Traditional dogma had considered the brain to be an inaccessible site, due to rudimentary studies in the late 19th century, and early 20th century with dyes injected into the blood not showing up in the brain upon autopsy^[12]^. Years later, an extension of this experimentally derived dogma also assumed that the CNS was among many tissues to be an “immunoprivleged” site^[13]^ largely derived from studies of grafts transplanted in the CNS that failed to be rejected when similarly grafted into other sites that were more immunologically accessible within the body. Additionally, the brain’s lack of draining lymphatics, the apparent immunoincompetence of microglia (the brain’s resident macrophages), and the assumption of CNS autoimmunity being a direct consequence from CNS antigen encounter by an immune cell cemented the idea of the brain being an inaccessible sanctuary away from the body’s immune system^[14]^. However, today this is not believed to be the case. A physiologically functioning BBB is now believed to act as a communication center of sorts, passing (and responding to) signals from the blood, regulating entry and exit of molecules from the blood and the CNS, and even changing as the somatic demands of the barrier changes^[15]^. This physiological barrier is often deregulated due to development of a brain malignancy such as glioblastoma, which again endorses the notion that tumors within the brain are inaccessible to the therapeutic agents as they cannot cross the BBB^[16]^. This fundamental change in understanding of how the BBB functions along with the rise of immunotherapy as a promising cancer treatment modality has opened wide the application of this therapy as a potential treatment for GBM^[17]^, which in the past has been extremely difficult to treat.

One specific modality of immunotherapy that has shown some promise in the treatment of GBM is the use of epigenetic modulators. Histone deacetylases (HDACs) play important roles in epigenetic changes and HDAC inhibitors as immunomodulatory agents have been useful in the preclinical arena to promote immune-mediated destruction of neoplastic cells in the CNS. These epigenetic modulators work to alter gene expression without alteration of the DNA sequences, through modulation of specific signaling cascades within the tumor^[18]^. In fact, one specific class of compounds that have currently shown promise in epigenetic modulation of GBM cells are the HDAC inhibitors^[19]^. This epigenetic approach towards cancer therapy involves tipping the balance between the activity of two different enzyme families, histone acetyltransferases (HATs) and HDACs. HATs have classically been involved in increasing gene expression, while HDACs have been associated with gene silencing. Mutations in HDAC enzymes have been linked to tumor development, due to the lack of inactivation of aberrant genes involved in the regulation of important cellular functions including cell proliferation, cell cycle regulation, and apoptosis^[20]^. Following the discovery of these dysregulated pathways in tumor cells, investigation into HDAC inhibitors has become an active area of research. Some of these agents had questionable efficacy when used as monotherapy against many human tumors, but when utilized in combination therapies with standard-of-care treatment regimens, they showed synergistic or additive effects^[21]^. In glioblastoma specifically, this treatment modality has demonstrated both induction of apoptosis and promotion of antitumor immunity^[22]^ providing a potential method of immunotherapy directed against glioblastoma.

In this review article, we seek to examine the current understanding of HDAC enzymes, describe progress in the development of HDAC inhibitors being used to treat glioblastoma, and report other potential immunomodulatory agents and immunotherapy modalities with a potential to be directed to glioblastoma. As unvaryingly lethal as this tumor is, the potential of novel therapeutic agents must not be overlooked in HDAC inhibitors because any new therapy may provide a new chance at remission for glioblastoma patients who are in desperate need of novel approaches towards fighting their malignant condition.

## HDAC ENZYMES

HDAC enzymes serve as some of the most important effectors of epigenetic changes in the human body. First isolated from a calf thymus extract^[23]^, HDACs were found to catalyze the removal of acetyl groups from lysine residues of both histone and non-histone proteins, thereby effecting transcriptional changes within the cells^[24]^. This function of histone deacetylation was suspected to be caused by a complex of multiple enzymes, but early chromatography studies were unable to differentiate the function of individual enzymes that made up this complex. However, this state of understanding changed significantly following the cloning of the first HDAC enzyme in 1996 (aptly described as HDAC1 in the literature)^[25]^. This began a wave of research publications fully describing these enzymes and their functions. Today, there are 18 different human HDAC enzymes divided into two separate families and four classes based on their similarities to their yeast enzyme counterparts [Table 1].

All 18 HDAC enzymes belong to either the HDAC family or the silent information regulator 2 (Sir2) family, with the human versions of these enzymes being further subcategorized into the classes based on their similarities in amino acid sequence. HDAC1, HDAC2, HDAC3, and HDAC8 are all class I proteins with sequence similarity to a yeast protein, which is called the reduced potassium dependency 3 (Rpd3). HDAC4, HDAC5, HDAC6, HDAC7, HDAC9, and HDAC10 are all class II proteins with sequence similarity to the yeast protein histone deacetylase-A 1 (Hda1). Class I HDACs are ubiquitously expressed in all tissues while class II HDACs are tissue-specifically expressed^[26]^. Sirtuin is a word coined from its founding member Sir2 in the yeast *Saccharomyces cerevisiae*. Sirtuin 1 (SIRT1), SIRT2, SIRT3, SIRT4, SIRT5, SIRT6, and SIRT7 in humans are all class III proteins with sequence similarity to the *S. cerevisiae* protein known as the Sir2. Finally, HDAC11 is the lone member of class IV and shares sequence similarity to both the class I and class II HDACs. These enzymes, for the most part, were numbered according to the order in which they were discovered.

**Figure 1.**
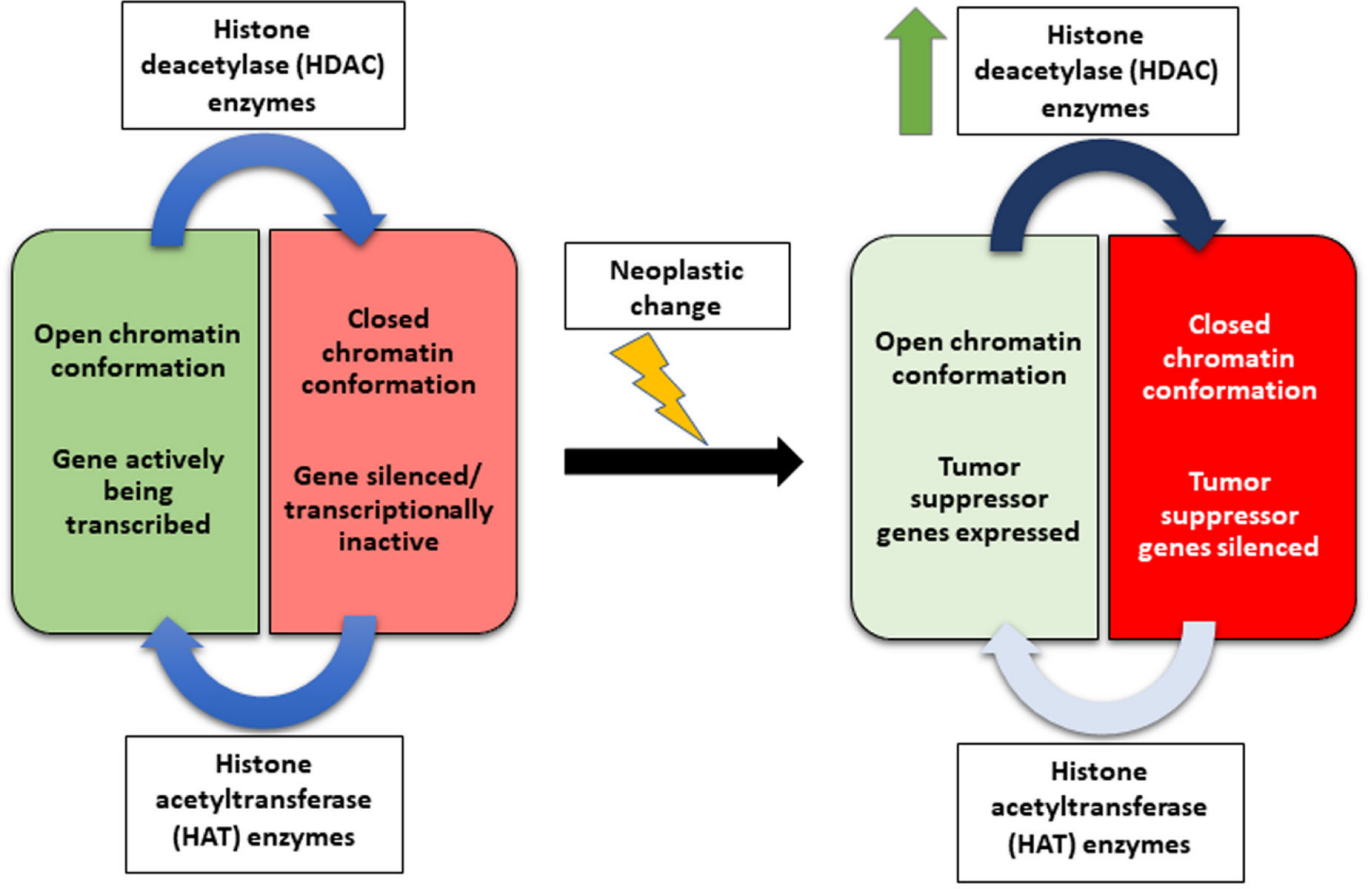
Histone acetyltransferase (HAT) and histone deacetylase (HDAC) in balance - physiologic *vs*. pathologic. In physiologic state, HAT enzymes and HDAC enzymes work in tandem to regulate gene transcription. HATs induce an open chromatin conformation (favoring gene transcription), which is counterbalanced by the action of HDACs that induce a closed chromatin conformation (favoring gene silencing). In pathologic state (e.g., neoplastic change) this balanced is tipped, favoring either an unregulated open chromatin conformation or an unregulated closed chromatin conformation. Schematically shown is an instance of an unregulated closed chromatin conformation due to a pathologic increase in HDAC enzymes. This unregulated, pathologic state may silence physiologic regulatory pathways in the cell, such as those protein products that regulate the cell cycle genes (e.g., tumor suppressor genes)

HDACs in classes I, II, and IV are in the superfamily of proteins known as the arginase/deacetylase superfamily, which contains the arginase-like amidino hydrolases and the HDACs. The HDAC enzymes in classes I, II, and IV belong to the classical HDAC family and require a zinc ion (Zn^2+^) for their catalytic action to take place. HDACs in class III, however, belong to the deoxyhypusine synthase-like nicotinamide adenine dinucleotide (NAD)/flavin adenine dinucleotide-binding-domain superfamily of proteins, which contain the Sir2 proteins as well as many other sequence-similar enzyme families. In contrast to the classical HDAC family of enzymes, class III enzymes require NAD^+^ as a cofactor for enzyme activity instead of a Zn^2+[27]^. While there are subtle differences in the classification scheme of these enzymes, they play an essential functional role in maintaining the balance between histone acetylation and deacetylation. This balance ultimately mediates access of transcriptional machinery to the chromatin of the cell, with downstream consequences such as alteration in gene transcription. However, functionality of these enzymes is much more complicated than one HDAC per one histone (or non-histone) protein. These enzymes as a superfamily are biologically essential due to their opposition of the effects of HAT enzymes, where a defect in this balance leads to epigenetic changes in the aberrant tissue [Figure 1].

A change in the cellular balance between HAT and HDAC enzyme activity modifies gene expression and translation of mRNA transcripts into protein products. However, this cellular balance is very delicate and has been classically shown that “minor” histone modifications can greatly influence gene transcription^[28]^. In fact, in one genome-wide mapping study, HDACs were observed to be bound to chromatin at actively transcribed genes, but not silent genes^[29]^. These HDACs are believed to be able to reset active chromatin, silencing the gene after making the desired protein product by the cell. Additionally, non-histone proteins are also subject to cellular changes through acetylation. Noteworthy non-histone proteins that can cause great cellular change include transcription factors, chaperone proteins, viral proteins, and proteins involved in DNA repair, recombination, and replication^[30]^. These non-histone proteins have been implicated in essential cellular processes such as chromatin remodeling, cell cycle regulation, apoptosis, autophagy, and actin nucleation^[31]^. The HDACs have been implicated in pathology as well, where their dysregulation halts the repression of active genes in the cell, leading to an abnormal expression of certain protein products. Alternatively, HDACs can also be overexpressed in abnormal tissue, leading to the silencing of regulatory genes [Figure 1]. Over the years, abnormal HDAC transcripts have been linked to multiple pathologies including neurological diseases, immune disorders, and a multitude of cancers^[32,33]^.

Cancer is a particular field where HDAC enzymes are heavily implicated, as there are correlations between somatic DNA mutations in histone-modifying enzymes and human malignancy^[34]^. One of the first examples of note was the discovery of a mutation in HDAC2, leading to microsatellite instability in those individuals with hereditary non-polyposis colorectal carcinomas^[35]^. The expression of HDAC transcripts has also been found to be variable in tumors when compared to normal somatic tissue, such that newer studies can link abnormal HDAC activity in 21 liquid and solid human tumors^[36]^. These changes in HDAC activity may lead to changes in histone acetylation status, thereby leading to increase in transcription of human oncogenes or suppression of tumor suppressor genes. Aberrant expression of HDACs has been shown to be correlated with a poor clinical prognosis^[37]^. These enzymes ultimately play an essential role in the body, providing a stabilizing force to the action of HATs and effecting epigenetic change. When researchers knew that these enzymes were often aberrantly expressed in tumors, they began setting their sights on understanding their roles in the pathogenesis behind one of the deadliest human cancers, glioblastoma.

## GLIOBLASTOMA AND DEREGULATION OF HDAC ENZYMES

HDAC enzymes may play a role in the tumorigenesis of glioblastoma through a yet-undetermined mechanism. HDACs are believed to be effectors of epigenetic changes observed in neoplastic tissue, particularly glioblastoma, when compared to non-neoplastic tissue. Among the many epigenetic alterations observed in glioblastomas, changes in HDACs specifically present an opportunity to monitor the transcriptional status of the genome in these tumors. Preliminary evidence showed that class II and class IV HDACs display decreases in mRNA expression in glioblastoma when compared to other, more low-grade gliomas and normal brain tissues, and an increased amount of acetylation in histone protein H3^[38]^. Histone modifications are frequent epigenetic changes observed in tumor analysis^[39]^. Additionally, one large-scale sequencing study of the protein-coding genes in glioblastomas revealed mutations in two genes *HDAC2* and *HDAC9*^[40]^. While this handful of preliminary studies have shown that HDAC enzymes see a decrease in expression, other more recent studies have shown HDAC enzymes seeing an increase in their expression, further complicating the picture of expression of HDAC enzymes in glioblastomas.

In recent years, further cytologic examinations of tumor samples have revealed an ambiguous picture as to the expression of HDAC enzymes in glioblastoma^[41]^. Looking at studies focusing on the expression patterns of HDACs in glioblastoma, these tissues seem to exhibit slightly and variably increased HDAC1, HDAC3, and HDAC6 expression levels as compared to non-neoplastic brain tissues examining both protein and mRNA within tissue samples^[42]^. The findings were further confirmed and even expanded to demonstrate that HDAC1 and HDAC3 expression levels correlated with WHO tumor grades, with the highest expression occurring in the most malignant gliomas. HDAC3, in particular, was correlated with poor survival. Another study observed that HDAC9 was overexpressed in glioblastomas with a poor prognosis^[43]^. The role of SIRT in glioblastoma is currently under debate due to equivocal findings across multiple studies. While many studies have correlated the down regulation of SIRT1 and SIRT6 in glioblastoma^[44,45]^, other studies have shown conflicting evidence as to whether the class II HDAC enzymes act as tumor suppressors or oncogenes^[46,47]^. The debate as to the role of class II HDACs will undoubtedly continue as the research into their roles becomes increasingly robust throughout the years. The classical HDAC family of enzymes is more clinically relevant as therapeutic agents have been developed to inhibit these aberrant enzymes. These therapeutic agents are currently undergoing clinical trials and are showing promise as potential new therapeutic modality for glioblastoma.

Another way to examine the HDAC expression in glioblastoma is to examine the effects and response displayed by these tumors when treated with HDAC inhibitors. Although this might be a more retrospective method of analysis and may be less clear due to the ambiguous mechanism of action of many HDAC inhibitors, this method may give some idea to which HDAC enzymes are aberrantly expressed in these tumors. We may be able to analyze the HDAC expression in tumor samples but the response of the tumor to a HDAC inhibitor as a potential therapy is a much more fruitful line of inquiry, emphasizing clinical results over cytologic curiosities. Ultimately, cytologic examination of glioblastoma tissue indicates which HDAC enzymes are aberrantly expressed, which goes on to inform which HDAC inhibitor may be useful for that tumor in particular, offering a potentially personalized approach to glioblastoma treatment. This review article will reveal that the answers, however, are not always clear-cut and highlight the complicated nature of these tumors, their protein expression, and their dysregulation leading to increased cell proliferation and malignant expansion.

## HDAC INHIBITORS

While biochemical investigation into HDAC enzyme activities was blossoming in the early 1970s, it was discovered in 1977 that millimolar concentrations of n-butyrate caused accumulation of acetylated histones^[48]^. It was subsequently confirmed that n-butyrate acted to inhibit histone deacetylation^[49]^. However, a direct causal relationship between these acetylated histones and n-butyrate was non-specific and unable to be verified, due to the documented effect of n-butyrate on cell membranes and many other enzymes other than HDAC. Later, the naturally occurring antifungal antibiotic trichostatin A (TSA) was discovered to be more potent for HDAC inhibition^[50]^. TSA, a hydroxamic acid compound, was found to inhibit cell cycle progression through direct inhibition of HDAC enzymes, thereby providing genetic evidence of a direct cellular target that TSA acted to inhibit fungal growth. A few years later, a fungal cyclic peptide known as trapoxin was also found to strongly inhibit HDACs, this time displaying an irreversible enzymatic inhibition^[51]^. These compounds served as a proof of premise, where HDACs could be inhibited with the use of exogenous compounds. However, these compounds had yet to find a clinical use.

In 1998, two later compounds to be clinically significant HDAC inhibitors were reported in the literature: suberanilohydroxamic acid (SAHA) also known as vorinostat and FK228 also known as romidepsin^[52,53]^. Phase I clinical trials of FK228 conducted at the National Cancer Institute confirmed that this compound was effective for the therapy of cutaneous and peripheral T-cell lymphoma. This finding stimulated the interest of many researchers and began increased development of HDAC inhibitors towards the treatment of multiple cancers. After years of drug development, SAHA (vorinostat) was the first HDAC inhibitor approved for use in cancer chemotherapy^[54]^ with FK228 following closely behind a few years later for approval in 2009. Multiple derivatives and novel compounds followed these two prototypic HDAC inhibitors, ultimately going on to have many investigational compounds being researched, all towards modifying the epigenetic expression in tumor cells through the inhibition of HDAC enzymes.

The HDAC inhibitors available today have wide variations in their function, structure, and mechanism. These inhibitors (similarly to their HDAC enzyme targets) can be divided into four classes on the basis of their chemical structure: hydroxamate, short-chain fatty acid (carboxylate), benzamide, and cyclic peptides [Table 2]. Adapted from recent investigations^[55,56]^ and clinical trial records from the National Institutes of Health, these agents and their various progress towards approval by the United States Food and Drug Administration (FDA) for use in glioblastoma has been compiled. The hydroxamic acid derivatives now include the compounds of azlaic bishydroxamic acid, m-carboxycinnamic bishydroxamic acid, dacinostat (LAQ824), a novel HDAC inhibitor known only as AR-42, panobinostat (LBH-589), quisinostat, and suberic bishydroxamic acid, among the already known compounds TSA and SAHA. Short-chain fatty acid derivatives include pivaloyloxymethyl butyrate (pivanex, AN-9), sodium butyrate, buphenyl (sodium phenylbutyrate), and valproic acid. Benzamides include the lone HDAC inhibitor entinostat (MS-275) and cyclic peptides still include the lone inhibitor of romidepsin. Miscellaneous agents displaying HDAC inhibitory activity include diallyl trisulfide (DATS) and tubacin. The above agents have shown clinical efficacy against many clinical entities but are most notable in their ability to be used in cancer chemotherapy.

The precise mechanism for which HDAC inhibitors ultimately cause an anti-cancer effect is not completely understood. These agents typically inhibit cancer cell proliferation through causation of cell cycle arrest, differentiation, and/or apoptosis. Studies show that all HDAC inhibitors activate either the extrinsic or intrinsic pathways of apoptosis in cancer models (when used in a combination therapy), with some activating both apoptotic pathways^[57]^. As we will discuss later, these agents have also been found to play an immunomodulatory role against tumor cells as well. Ultimately, the mechanism for which these HDAC inhibitors exert their cellular changes does not need to be completely understood to observe clinical changes and the promise of these novel therapies. Some of these agents have already been approved for use and are in multiple phases of clinical trials towards the treatment of many pathologies [Table 2]. However, none of these agents have yet been approved for clinical use in the treatment of glioblastoma, a tumor that is in desperate need of novel therapeutics due to its dismal 5-year survival rates.

## HDAC INHIBITORS FOR ANTITUMOR EFFECTS IN GLIOBLASTOMA

Glioblastoma, as one of the deadliest human neoplasms with few effective treatment options, has frequently been a target of new treatment modality through clinical trials. HDAC inhibitors are no exception to this and these inhibitors have undergone multiple clinical trials to test their efficacy in glioblastoma. These agents have displayed both pre-clinical efficacy in their use, as well as efficacy in clinical use either as monotherapy or in combination regimens^[55]^. Ultimately, there is a more vested interest in the clinical outcomes and efficacy, but in order for these clinical trials to be well reasoned there must be a strong research base and rationale behind the use of HDAC inhibitors.

There is a two-fold rationale for the use of HDAC inhibitors in glioblastoma therapy. First, HDAC inhibitors promote a more open chromatin conformation in the tumor cells and thereby permit the DNA alkylating chemotherapeutic agents (e.g., TMZ) to access genomic DNA and increase the sensitivity of the tumor cells for these agents. Second, HDAC inhibitors help reverse some of the abnormal genetic silencing in glioblastoma, where it is presumed that this will lead to enhanced cell-cycle arrest and apoptosis from the action of DNA damaging agents^[58]^. SAHA plays a unique role as an HDAC inhibitor that acts as a pan-inhibitor of all HDAC enzymes, while other HDAC inhibitors are more specific in their action. All the HDAC inhibitors, however, seem to cause increases in acetylation in histone and non-histone proteins and reactivate p21Waf1/Cip1, a protein that contributes to cell-cycle arrest due to its role as a tumor suppressor protein^[59]^. Traditionally, it has been believed that all HDAC inhibitors have difficulty in penetrating the BBB at low doses and require high doses for therapeutic effects. Some selective HDAC inhibitor classes such as the fatty acids^[60]^ and benzamide compounds^[61]^, however, have shown increased penetration into the BBB on imaging studies. Interestingly enough, it also seems that there is some selectivity between HDAC inhibitors affecting tumor cells *vs*. normal cells. One older study, in particular, found that the antitumor effects of hydroxamate-containing HDAC inhibitors displayed antitumor selectivity and did not affect somatic cells^[62]^, apprising the possibility of a safe agent with few toxicities to normal cells. Additionally, HDAC transcripts have been observed to be both increased and decreased in tumor cells undergoing exposure to HDAC inhibiting agents^[63]^. The results showed a lack of clear-cut cell cycle arrest effect, which the researchers recognized during other pre-clinical studies. The lack of specificity on HDAC substrates by HDAC inhibitors presents a mechanistic grey area concerning the use of HDAC inhibitors in glioblastoma specifically.

HDAC inhibitors have also shown efficacy in the preclinical arena towards the chemotherapy of GSCs. Targeting GSCs in particular is a major therapeutic undertaking as these cells often form the seeds of recurrence for glioblastoma after initial therapy and also confer resistance to previously used standard-of-care therapeutic agents. One study showed that the HDAC inhibitors TSA and valproic acid significantly reduced proliferation rates of GSCs by decreasing the amount of neural and embryonic stem cell surface markers expressed by these cells, indicating that these HDAC inhibitors stimulated differentiation in GSCs^[64]^. The HDAC inhibitor SAHA also demonstrated capabilities of slowing down tumor proliferation and triggering autophagy in GSCs, rather than induction of differentiation seen with TSA and valproic acid^[65]^. HDAC inhibitors have also been implicated for use in combination therapies against GSCs. Another study demonstrated that combination of the HDAC inhibitors SAHA, valproic acid, and sodium phenylbutyrate when used in combination with the FDA-approved proteasome inhibitor bortezomib caused high cytotoxicity against GSCs in cultures^[66]^. Specific chemotherapy that targets GSCs is in high demand as effective treatments for recurrent glioblastoma shows very poor efficacy. At least in the preclinical arena, HDAC inhibitors have demonstrated their efficacy in targeting GSCs in particular either through monotherapy or in combination with other known therapies.

Regarding current clinical trials under way for each specific HDAC inhibitor towards the treatment of glioblastoma, many HDAC inhibitors have shown considerable clinical promise but have yet to be approved by the FDA. These agents are said to be in the pre-clinical phase, where there are multiple rationales for specific inhibitors. Beginning with the examination of the hydroxamate derivative compounds, SAHA (vorinostat) has been shown *in vitro* to inhibit cell proliferation in glioblastoma cell lines independent of their p53 status, leading to an accumulation of cells arrested in the G2/M phase of the cell cycle, increased expression of anti-proliferative genes, and decreased levels of pro-growth genes^[67]^. SAHA additionally induces differentiation, apoptosis, and autophagy in human glioblastoma cell lines. As mentioned earlier, TSA is another hydroxamate compound akin to SAHA in HDAC targets. Similar to SAHA, TSA also induces differentiation and apoptosis in human glioblastoma cell lines, resulting in a higher expression of astrocytespecific markers [i.e., glial fibrillary acidic protein (GFAP)] and reduced expression of vimentin and nestin (common markers of neuro-epithelial stem cells)^[68]^, increasing the recognizability of the tumor cells to the immune system. Of the short-chain fatty acid HDAC inhibitor class, valproic acid has been found to exhibit its antineoplastic effects through decreasing the activity and expression levels of matrix metalloproteinases (MMPs) in addition to the inhibition of activity of HDAC class I and II, thereby decreasing the invasiveness of glioblastoma cell lines^[69]^. Phenylbutyrate, another short-chain fatty acid HDAC inhibitor, has demonstrated its efficacy (like TSA) through increasing the expression of GFAP in human glioblastoma cells in culture as well as redistributing intracellular GFAP thereby enhancing gap junction communication between tumor cells through upregulation of the protein connexin 43^[68,70]^. Entinostat, the lone benzamide HDAC inhibitor, has been shown as a promising compound in the treatment of glioblastoma through its ability to significantly reduce cell growth, upregulate the cell cycle inhibitor p21Waf1/Cip1 and induce cell cycle arrest in the G0/G1 phase, and induce apoptotic cell death in glioblastoma cell lines^[71]^. Entinostat has also been shown to have some immunomodulatory roles similar to TSA through regulation of production of cytokines and inhibiting Treg cells in certain cancer models^[72]^. Romidepsin, the lone cyclic peptide HDAC inhibitor, has been shown at nanomolar levels in glioblastoma cell lines to cause inhibition of cell proliferation and induction of apoptosis (through the increased expression of the cell cycle inhibitor p21Waf1/Cip1 and the pro-apoptotic protein Bad and the decreased expression of the anti-apoptotic proteins Bcl-xL and Bcl-2)^[73]^. Finally, of the two miscellaneous HDAC inhibitors, DATS has been shown to cause upregulation of the cell cycle inhibitor p21Waf1/Cip1 and the tumor suppressor p53 in order to cause cell cycle arrest in glioblastoma cells and is unique in that it is derived from garlic and demonstrates less toxicity to normal cells than other HDAC inhibitors^[74]^. Tubacin, the other miscellaneous HDAC inhibitor, is a specific for HDAC6^[75]^ and it has been proposed to be useful because HDAC6 is known to be increased in certain high-grade gliomas. All the above HDAC inhibitors have shown considerable promise for growth inhibition in glioblastoma, but only a few of these agents have made it into the clinical trials as of now.

Only a few selected HDAC inhibitors that have shown promise in the pre-clinical realm translate so seamlessly over to show efficacy in the clinical realm. Vorinostat, romidepsin, and valproic acid are particularly notable to have seen translational promise in the preclinical realm as well as in the clinical realm. Vorinostat as a monotherapy progressed through multiple Phase I and Phase II trials, with the results of one Phase II trial indicating that it was well tolerated in recurrent glioblastoma patients, and its efficacy was seen to extend life by a few months in a subpopulation of those with recurrent glioblastoma^[76]^. Romidepsin also went through both Phase I and Phase II trials but had disappointing outcomes in progression free survival with the conclusion that although the drug demonstrated success in the preclinical arena, when used in clinics an inadequate amount of the drug reached the actual tumor in the CNS^[77]^. However, this agent showed success when used in combination therapies. Valproic acid similarly showed success in clinical trials, but only when used in combination therapies and not when used as a monotherapy^[78]^. In fact, many other HDAC inhibitors listed in Table 2 in various trials for use in glioblastoma are in combination therapies and may yet show results when combined with the standard-of-care agents. However, HDAC inhibitors when used as monotherapy have yet to yield the progression free survival results that the preclinical mechanistic evidence would suggest, with an exception to vorinostat (and even then, only modestly so). To understand the full picture of these promising new agents, one must look at both the preclinical and the clinical data housed in trials. Unfortunately, there seems to be a wealth of new mechanisms to be revealed and understood as to the biological pathways these agents are inhibiting. A more profound understanding of glioblastoma pathogenesis and the associated aberrant pathways inhibited by these agents is essential to translate the benefits from the preclinical bench to the clinical arena.

## HDAC INHIBITORS FOR ENHANCING ANTITUMOR IMMUNITY IN GLIOBLASTOMA

While there have been a variety of preclinical studies regarding the effects of HDAC inhibitors specifically in glioblastoma, one of the most interesting effects is alteration of the tumor itself to increase tumor susceptibility to antitumor immune attack. Many cells of the immune system act as surveillance cells, effectively patrolling the body to eliminate neoplastic cells as soon as they are found^[79]^. However, many tumors are notorious for down regulating these markers on their surfaces, effectively “hiding” from the immune system to evade elimination and continue their unfettered growth. While there are many important cell types (microglia, T cells, *etc*.) involved in the surveillance of the body’s somatic tissues for signs of pathological changes, one of the cell types most important to the preclinical mechanism of HDAC inhibitors and antitumor immunity are NK cells. These cells act to “check” or surveille surface proteins displayed on the exterior of many cells, checking these cells for signs of stress, infection, or neoplastic change. If an NK cell finds a cell within the body that has undergone one of these pathological changes, the NK cell releases cytotoxic granules containing toxic compounds known as perforins and granzymes, which act synergistically to induce apoptosis in the target cell. These NK cells have been described in the literature to be the agents that lyse GBM cells as they are recognized during their surveillance, with HDAC inhibitors playing a role in upregulating the surface markers that help to mark these malignant cells as a target for elimination^[19,22]^.

**Figure 2.**
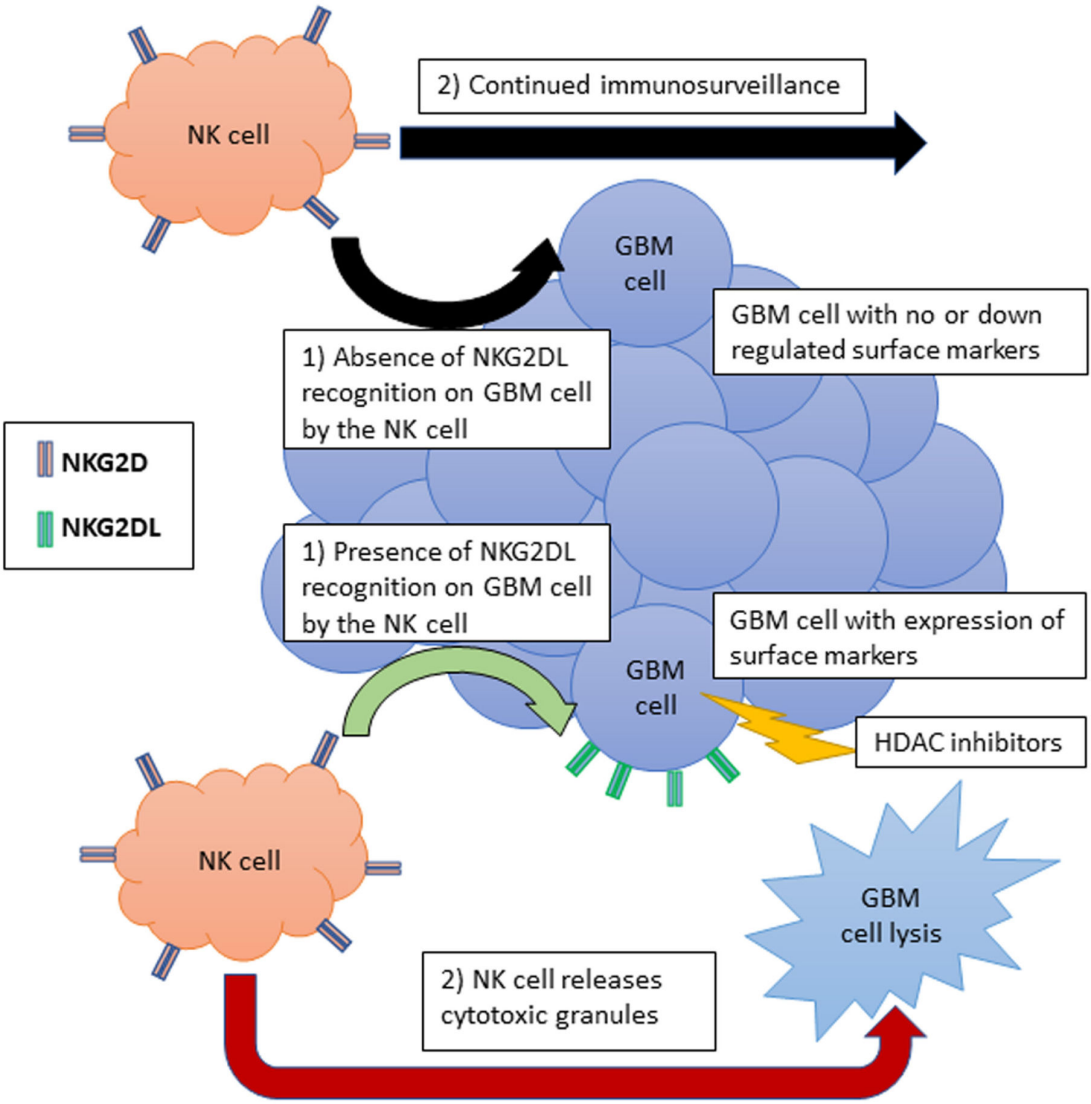
Natural killer (NK) cell antitumor immunity under histone deacetylase (HDAC) inhibitor influence. A tumor such as glioblastoma multiforme (GBM) is able to eschew immune surveillance by NK cells through either down regulation of surface marker [i.e., natural killer group 2D ligand (NKG2DL)] or through the activation of matrix metalloproteinases to degrade surface marker once they reach the tumor cell’s surface. Some selected HDAC inhibitors such as trichostatin A have been shown to upregulate surface markers in GBM. This upregulation of surface markers on the tumor cell’s surface makes the tumor able to be recognized by the immune system (through binding of natural killer group 2D to NKG2DL), causing the NK cells to release cytotoxic granules and leading to apoptosis in the GBM cell

The NK cells possess a constitutively expressed receptor on their surface known as natural killer group 2D (NKG2D) that is essential for recognition of abnormal human tissues^[80]^. This receptor recognizes a ligand known as natural killer group 2D ligand (NKG2DL) that is expressed by somatic cells in times of stress, marking them for destruction via release of cytotoxic granules from NK cells^[81]^. However, GBM cells have found a mechanism for evading this natural antitumor immunity through the down regulation of NKG2DL, thereby avoiding surveillance, or through the activation of MMPs that act to shed the natural expression of these ligands and release them into the microenvironment surrounding the tumor^[82]^. Interestingly enough, it is now known that expression of this specific ligand in GBM cells is regulated by HDAC enzymes, where overexpression of these enzymes in tumor cells is effectively silencing the genes responsible for the expression of these surface markers [Figure 2]. HDAC inhibitors have therefore been shown to induce the expression of these ligands on the surface of GBM cells, thereby allowing these cells to be recognized by the immune system and subsequently be destroyed^[83]^.

Other leukocytes that have been implicated in antitumor immunity among NK cells also include Treg cells and microglia. Instead of priming the tumor cells for removal by the immune system, current inquiry has looked into the role of these leukocytes in the tumor microenvironment, and how their inhibition may increase the tumor’s susceptibly to clearance by the immune system^[84]^. One pilot study in particular looked at lymphodepletion of Treg cells through the use of monoclonal antibodies in those with glioblastoma and showed enhanced antitumor immunity, as it had been shown in the past that these Treg cells were associated with immunosuppression of glioblastoma^[85]^. Depleting the Treg cells through the use of the anti-CD25 monoclonal antibody daclizumab was able to paradoxically enhance antitumor immunity. Additionally, another study looked at using anti-PD-1 and anti-CTLA-4 antibodies for the use of inhibiting Treg cell function as well, which showed improved survival in mouse models^[86]^. Microglia have been similarly targeted to enhance antitumor immunity, as they have increased presence within the GBM microenvironment and are assumed to have roles in local immunosuppression. Inhibition of the signal transducer and activator of transcription 3 (STAT3) pathway within tumor cells has shown improved outcomes in mouse models specifically, with one study using the siRNA-based method to activate these cells within the tumor microenvironment and subsequently slow tumor growth^[87]^. Another study showed success using the same rationale but utilizing the miR-124 inhibition of the STAT3 pathway to enhance antitumor immunity^[88]^. While these studies have demonstrated promising concepts for future investigation regarding antitumor immunity in leukocytes, these effects are largely limited to the tumor microenvironment and the biggest successes have only been demonstrated in mouse models or had a very small sample size. Additional investigation is obviously required before these potential therapeutic modalities are ready for human trials.

Of the known HDAC inhibitors, TSA seems to show promise in the preclinical realm for enhancing antitumor immunity; but unfortunately, when brought into the clinical arena, TSA showed high toxicity and low efficacy. While this compound has been shown to upregulate NKG2DL expression on GBM cells directly, it is unclear whether this action is due to epigenetic transcriptional alteration within the tumor cell or this is due to reduction of secretion of MMPs^[69]^. The immunostimulatory effect of TSA was shown to be also dependent upon the presence of NK cells, as evident from the use of an anti-NKG2D antibody significantly reducing the amount of observed GBM cell lysis *in vitro*. While this compound showed considerable preclinical promise, its high toxicity and low efficacy has made other HDAC inhibitors such as vorinostat, romidepsin, and valproic acid as more promising candidates for potential future monotherapy in GBM. These HDAC inhibitors unfortunately do not display the same antitumor immunity as other HDAC inhibitors in the preclinical arena but are the most likely candidates to be used for future monotherapy or combination therapy in clinical trials.

While HDAC inhibitors have been used to treat cancers successfully in the past and have seen modest success in their use against GBM specifically, this is the first time that these agents have been utilized as an immunotherapy regimen in GBM. As it has already been described in this article, while an agent may see mechanistic success in the laboratory setting this may or may not translate to the clinical realm through the process of FDA approval and clinical trials. Studies such as these offer exciting possibility of new therapeutic modalities for a formidable clinical challenge that is in desperate need of innovation.

## IMMUNOTHERAPY IN CONTROLLING GROWTH OF GLIOBLASTOMA

One of the most exciting new therapy modalities being examined for the treatment of glioblastoma is immunotherapy and immunomodulation, or harnessing/modifying the body’s immune system to help fight the tumor directly. However, while in theory these therapies may be very promising, in practice the tumors themselves have multiple mechanisms of immunosuppression that lead to promising *in vitro* results, but further studies do not necessarily see the same *in vivo* or clinical results^[89]^. These tumors cause systemic immunosuppression through their release of cytokines, which inhibit lymphocyte proliferation and promote production of the well-characterized immunosuppressive factors such as transforming growth factor-β, interleukin-10, prostaglandin E2, and gangliosides^[90]^. These tumors also secrete the chemoattractants such as monocyte chemoattractant protein-1, colony stimulating factor-1, granulocyte/macrophage colony stimulating factor-1, and hepatocyte growth factor to recruit microglia to the local tumor microenvironment in order to support tumor cell proliferation and tumor growth, as well as secrete factors that lead to local immunosuppression and inhibition of the remaining immune system cells that are now unavailable to attack this tumor^[43]^. Finally, these tumors also have immunosuppressive antigens on their cell membrane surfaces and secrete factors that lead to further inhibition of the immune system from properly attacking these tumors^[91]^. With these mechanisms in place in glioblastomas, it becomes essential to first understand the immunosuppressive mechanisms employed by these tumors before delving into the immunotherapy/immunomodulation mechanisms that have been showing such preclinical promise and possibly to explain the lack of translation of this promise into the clinical realm.

Despite the immunosuppressive action inherent in glioblastoma, this tumor has been the subject of multiple studies using multiple immunomodulatory methods besides HDAC inhibitors. One of the more exciting strategies is the use of “trained” T cells directed towards known tumor antigens, also known as CAR-T cell therapy. This therapy modality has been applied towards glioblastoma, with mixed results for a variety of reasons. These barriers to successful therapy include the previously-discussed barriers to cellular delivery and immunosuppressive microenvironment as well as proper selection of appropriate glioblastoma antigens to train the T cells^[10]^. However, a recently published high-profile case study has shown regression of recurrent GBM following the use of this CAR-T cell therapy^[92]^, heralding this particular treatment modality as extraordinarily effective in certain cases and in certain tumor types. Ultimately, this treatment modality shows considerable promise and with initial Phase I trials suggesting that this therapy is safe without dose-limiting side effects, this strategy will be very likely to continue to be considered as our lists of GBM antigenic targets as well as continue to increase as our understanding of these tumors becomes more robust^[93]^.

**Figure 3.**
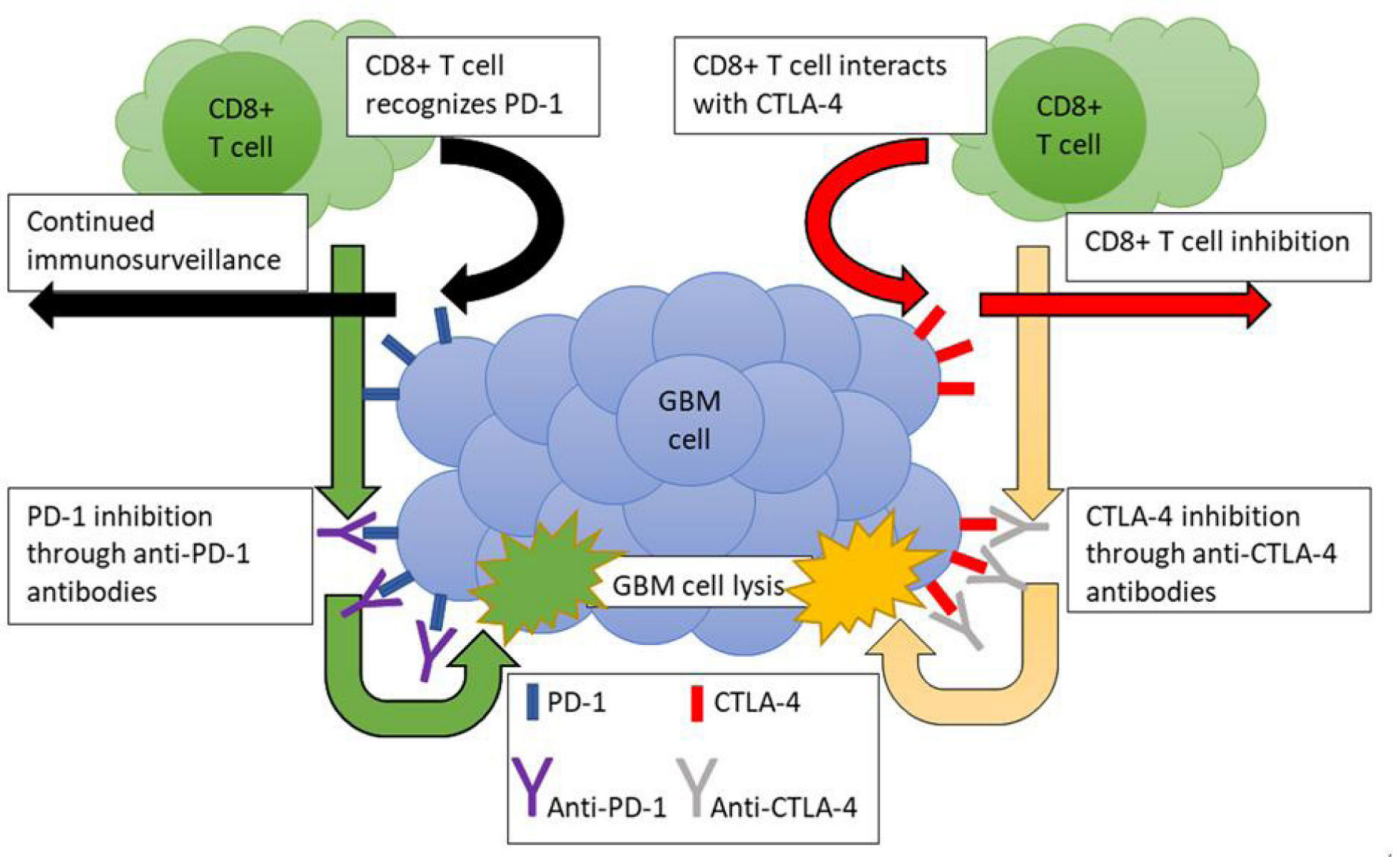
Potential immunotherapy for glioblastoma multiforme (GBM) using anti-programmed death-1 (PD-1) and anti-cytotoxic T lymphocyte associated antigen-4 (CTLA-4) antibodies. Anti-PD-1 and anti-CTLA-4 antibodies have been utilized in different human malignancies to promote antitumor immunity with enormous success in selective cases. This antitumor immunity is proposed to be mediated through disinhibition/stimulation of cytotoxic T cells for eliminating the malignant cells. Anti-PD-1 inhibits the interaction of programmed death-ligand 1 on cytotoxic T cells, making the T cells believe that the cell they are interacting with is foreign. Similarly, anti-CTLA-4 inhibits the interaction of the inhibitory CTLA-4 surface marker with B7 surface marker of the cytotoxic T cell, allowing for recognition of the malignancy by the T cells. The cytotoxic T cells then release their cytotoxic granules, leading to apoptosis in the GBM cells

Another immunomodulatory treatment modality that has shown promise in recent years is the use of immune checkpoint inhibitors, which are agents that help “unblock” the regulation induced by tumor cells on the immune system, priming the tumor cells for killing. Specific immune checkpoint proteins that have been investigated for immunotherapy of GBM include: PD-1/PD-L1, CTLA-4, T cell immunoglobulin and mucin containing protein-3, and indoleamine-(2,3)-dioxygenase^[11]^. The rationale behind these therapies involves the use of monoclonal antibodies designed to target these surface markers in order to increase the tumor’s susceptibility to immune attack by cytotoxic T cells [Figure 3]. These immune checkpoint proteins restrain immune responses and thereby prevent T cells from killing the tumor cells. When these proteins are gridlocked with monoclonal antibodies, the restraints on the immune responses are released and T cells turn into weapons to kill tumor cells. Specific to glioblastoma, these therapies have been explored as a promising crop of new therapeutic targets^[94]^. While these targets have shown promise in clinical trials, the ultimate assessment of these agents are mixed at best. Each of these agents has been speculated to be a useful therapeutic modality when combined with other chemotherapy, radiation, or with other immunomodulatory treatments^[95]^. Unfortunately, these strategies have yet to show the promising results in the clinical realm.

Finally, GBM is the target of yet another immunomodulatory treatment modality, the use of vaccine therapy to prime the immune system to fight the tumor directly and recognize recurrences, much in the same way our immune system already does with many infectious agents. These strategies have utilized multiple targets in an attempt to activate the immune system in a way where it is able to eradicate the tumor, which include: peptide vaccines, polyvalent dendritic cell vaccines, and heat shock protein vaccines. Again, akin to many other agents discussed in this article, these agents have shown mixed results depending on which clinical trial you examine and have been only suggested to supplement the already established standard-of-care treatments^[96]^. The movement for vaccine strategies for the treatment of GBM allows for considerable targeted therapy opportunities, with examination of personal tumor elements and vaccines that have been shown to effectively combat those tumors in the clinical arena from past studies as well in combination with existing standard-of-care regimens^[97,98]^.

Vaccines will continue to evolve as our understanding of tumor immunology continues to evolve, which is the crux of a comment on the progress in this certain field. Our understanding of tumor immunology is quickly expanding, and we are bringing into relief the degree of complexity in the tumor microenvironment. Still significant barriers to overcoming tumor-mediated immunosuppression, treatment delivery in the CNS, and proper selection of the correct targeted therapy are just a few of the limitations this therapy modality must overcome. However, a more profound mechanistic understanding of these tumors and more data regarding the efficacy of the immunotherapy treatment modality are showing promise. Perhaps immunotherapy for glioblastoma will become the panacea as it has been promised, despite the considerable work that must be undertaken and continued to reach such a horizon^[99,100]^.

## CONCLUSION

While glioblastoma continues to present a formidable preclinical challenge for researchers, further inquiry into the molecular pathogenesis, aberrant cellular pathways, and tumor immunology will ultimately aid in the development of more targeted therapies for a clinical entity that has yet to find a solution. Success in treating a disease with such a dismal survival rate will come from a well-rationalized approach that will translate into real-world clinical measures such as progression free survival. HDAC inhibitors are another promising treatment modality being investigated to combat this insidious malignancy. While these therapies may show promise, the mechanistic minutiae of why a therapy may or may not be effective is just as valuable. Continued work is required in the field of glioblastoma, as the promise that has been shown by these agents is begging to be brought to its fullest potential and may yet offer hope to those diagnosed with an illness long surrounded by pessimism, dread, and anxiety.

## Figures and Tables

**Table 1.** Characteristics of the human histone deacetylase enzymes and their similarity to yeast proteins

HDAC enzyme class	HDAC enzymes*	Protein family	Required catalytic cofactor	Resembled yeast protein sequence
I	HDAC1, HDAC2, HDAC3, and HDAC8	Histone deacetylase	Zn^2+^	Rpd3
II	HDAC4, HDAC5, HDAC6, HDAC7, HDAC9, and HDAC10	Histone deacetylase	Zn^2+^	Hda1
III	SIRT1, SIRT2, SIRT3, SIRT4, SIRT5, SIRT6, and SIRT7	Sir2 regulator	NAD^+^	Sir2
IV	HDAC11	Histone deacetylase	Zn^2+^	Class I and II HDACs

* HDAC enzymes have been divided into four classes based on their similarity in sequence and function to well-described yeast proteins. Class I enzymes include HDAC 1, 2, 3, and 8 that belong to the classical HDAC family, require a Zn^2+^ for their catalytic action, and are similar to the yeast protein Rpd3. Class II enzymes contain HDAC 4, 5, 6, 7, 9, and 10 that also belong to the classical HDAC family, also require a Zn^2+^ for their catalytic action, and are similar to the yeast protein Hda1. Class III enzymes differ most significantly from their HDAC counterparts, containing SIRT 1, 2, 3, 4, 5, 6, and 7 that belong to the distinct Sir2 regulator family, require NAD^+^ as an essential catalytic cofactor, and are similar to the yeast protein Sir2. Finally, class IV contains only HDAC11 that is also part of the classical HDAC family, requires a Zn^2+^ for its catalytic action as well, and most resembles the class I and II HDAC enzymes. These enzymes are numbered in the order in which they were discovered. HDAC: histone deacetylase; SIRT: sirtuin

**Table 2.** Histone deacetylase enzyme inhibitor classes

HDAC inhibitor class	HDAC inhibitor(s)*	HDAC target	Clinical trial in GBM	Clinical trial for other uses
Hydroxamic acid	ABHA m-Carboxycinnamic CBHA LAQ824 AR-42 Panobinostat Quisinostat SBHA TSA Vorinostat Belinostat	HDAC classes 1, II, and IV	Panobinostat in Phase II Belinostat in Phase II SAHA in Phase III	AR-42 in Phase I (acute myeloid leukemia) Panobinostat in Phase III (several cancers) Quinostat in Phase II (T-cell lymphoma) Vorinostat in Phase III (cutaneous T-cell lymphoma and other cancers) Belinostat indicated for use in treatment of peripheral T-cell lymphoma
Short-chain fatty acid	Pivanex Sodium butyrate Buphenyl Valproate	HDAC classes 1 and II	Buphenyl in Phase II Valproate in Phase II	Pivanex in Phase II (non-small cell lung cancer) Sodium butyrate in Phase II (endogenous antibiotics in gut) Buphenyl indicated for use in treatment of urea cycle disorders Valproate indicated for use in treatment of epilepsy, anorexia nervosa, panic attack, and anxiety disorders.
Benzamide	Entinostat	HDAC1, HDAC2, and HDAC3	Not available	Entinostat in Phase III (breast cancer)
Cyclic peptide	Romidepsin	HDAC1, HDAC2, HDAC3, and HDAC8	Phase I/II	Romidepsin indicated for use in treatment of cutaneous T-cell lymphoma and in Phase trials for many other cancers
Other	DATS Tubacin	Unknown for DATS HDAC6 for Tubacin	Not available	Not available

* HDAC inhibitors have been divided into four classes based on chemical makeup and HDAC classes they inhibit. Hydroxamic acid derivatives are some of the most well-described HDAC inhibitors and inhibit the classical HDAC family of enzymes. Pabinostat, bellinostat, and SAHA are all at the clinical trial phase of development for use in GBM, with numerous other compounds showing efficacy in clinical trials for other tumors. Short-chain fatty acid HDAC inhibitors are also relatively well described and inhibit class I and II HDAC enzymes. Buphenyl and valproate are both in the clinical trials for use in GBM with numerous other compounds showing efficacy in clinical trials for other tumors. Entinostat is the sole benzamide derivative HDAC inhibitor and it has been shown to inhibit class I HDAC enzymes. This compound has not yet been used in clinical trials for treatment of GBM but has gone to a phase III clinical trial for treatment of breast cancer. Romidepsin is the sole cyclic peptide derivative HDAC inhibitor and it has also been shown to inhibit class I HDAC enzymes. This compound has gone to phase I and II clinical trials for use in GBM and it has been approved for treatment of cutaneous T-cell lymphoma. Finally, DATS and tubacin are miscellaneous HDAC inhibitors that are currently under investigation and they have variable effects on specific HDAC enzymes. HDAC: histone deacetylase; ABHA: azlaic bishydroxamic acid; CBHA: carboxycinnamic bishydroxamic acid; SBHA: suberic bishydroxamic acid; TSA; trichostatin A; DATS: diallyl trisulfide; GBM: glioblastoma multiforme
